# Treatment of C-section diverticula with hysteroscopic resection in women without childbearing intention: a retrospective cohort study

**DOI:** 10.1186/s12905-020-00928-4

**Published:** 2020-04-21

**Authors:** Hui Shi, Jingyan He, Yunhe Gao, Shuang Qin, Jiaying Fan, Qing Xiao, Kuanrong Li, Huiying Liang

**Affiliations:** 1 Institute of Pediatrics, Guangzhou Women and Children’s Medical Center, Guangzhou Medical University, 9 Jinsui Road, Zhujiang Newtown, Tianhe District, Guangzhou, 510623 China; 2 Department of Obstetrics & Gynaecology, Guangzhou Women and Children’s Medical Center, Guangzhou Medical University, Guangzhou, China

**Keywords:** Cesarean section diverticulum, Cohort study, Hysteroscopic, Prognostic factor

## Abstract

**Background:**

Diverticulum, one of the long-term sequelae of cesarean section, can cause abnormal uterine bleeding, dysmenorrhea and chronic pelvic pain. Hysteroscopic resection of diverticula is thought to reduce abnormal uterine bleeding and chronic pelvic pain. In this study, we aim to describe the improvement after hysteroscopic resection of cesarean section diverticula (CSD) in women without childbearing intention, and to explore the variables associated with poor prognosis.

**Methods:**

A retrospective cohort study of women aged 25–48 with CSD diagnosis by transvaginal ultrasonography (TVS) and hysteroscopy that were enrolled at Guangzhou Women and Children’s Medical Center between June 2017 and December 2018. A total of 124 women met the inclusion criteria and all patients had undergone hysteroscopic resection and accepted a follow-up interview at the 3rd and 6th months postoperatively to record symptom improvement.

**Result:**

The mean of intraoperative blood loss and operative time of hysteroscopic resection were (12.94 ± 12.63) ml and (33.63 ± 6.87) min in 124 patients. Overall observed improvement rates of CSD symptom were 47.2 and 65.6% in the first 3 and 6 months, respectively. Multivariable logistic regression models revealed that timing of surgery < 14 days was a good prognostic factor associated with both 3-month improvement (OR, 16.59; 95% CI, 2.62–104.90; *P* = 0.003) and 6-month improvement (OR, 15.51; 95%CI, 1.63–148.00; *P* = 0.02); Patients with numbers of cesarean section (CS) ≥2 had a lower rate of improvement after 6 months of CSD repair surgery compared with patients who underwent one CS (OR, 8.29; 95%CI, 1.05–65.75; *P* = 0.04).

**Conclusions:**

A hysteroscopic repair might be an appropriate method for CSD in women who no childbearing intentions. The timing of surgery and the number of CS seems to be factors influencing the postoperative improvement of CSD.

## Background

Owing to the growing Caesarean section (CS) rate, the incidence of cesarean section diverticulum (CSD), a common complication of CS, is expected to increase as well [1]. CSD is defined as deficient uterine scars or scar dehiscence following a cesarean section, which involves the myometrial discontinuity at the cesarean scar [2]. In a meta-analysis of 21 studies involving nearly 2500 participants, the prevalence of CSD varies between 56 and 84% [3]. Although a clear definition of CSD is still the subject of some debate, abnormal uterine bleeding, prolonged menstrual bleeding, dysmenorrhea and chronic pelvic pain are generally accepted as the common symptoms [4–7]. These symptoms can seriously affect patients’ quality of life. Furthermore, the clinical implications of CSD due to possible CS scar ectopic pregnancy and a potentially higher risk of uterine rupture are of concern to obstetricians and gynecologists [1].

At present, the treatment of CSD includes hormonal contraceptive therapy and surgery. However, oral contraceptives can only result in a temporary improvement in symptoms [8, 9]. Thus, several surgical therapies have been developed including laparoscopic or hysteroscopic defect resection and vaginal defect repair [10]. Hysteroscopic resection is considered the least invasive among these operations, so far only a few studies reported on a hysteroscope resection of the CSD. Among them van der Voet et al. systematically reviewed the available literature and concluded that sample sizes, follow-up, and methodological quality of the selected papers were insufficient to draw solid conclusions [10].. Before hysteroscopic resection is implemented in women that suffer from postmenstrual spotting based on a CSD, better assessment of the effectiveness of the treatment is needed.

In this study, our aim is to describe the improvement after hysteroscopic resection of CSD and to explore the variables associated with poor prognosis.

## Methods

### Study population

The protocol of this retrospective cohort study was approved by the ethics committee of the Guangzhou Women and Children’s Medical Center between June 2017 and December 2018 (No. GO-2017-017). The requirement to obtain informed consent was waived because of the retrospective nature of the study, but an oral consent was obtained from some subjects at the time of telephone follow-up after full explanation of the purpose and nature of the procedure used.

All the enrolled patients fulfilled the following criteria: (1) history of at least one CS; (2) no childbearing intention; (3) residual myometrial thickness (RMT) at least 3 mm [11]; (4) diagnosed with CSD. by transvaginal ultrasonography (TVS) and hysteroscopy [12], and manifested clinical symptoms including prolonged menstrual, increase in menstrual volume, abdominal pain or irregular bleeding. Exclusion criteria included: (1) irregular menstrual cycle before CS; (2) previous intrauterine contraceptive device localization; (3) coagulation disorders; (4) abnormal uterine bleeding resulted from other organic uterine pathology, e.g., endometrial hyperplasia/polyps, malignancy, or submucosal myomas.

### Diagnosis and treatment of CSD

**Fig. 1:**
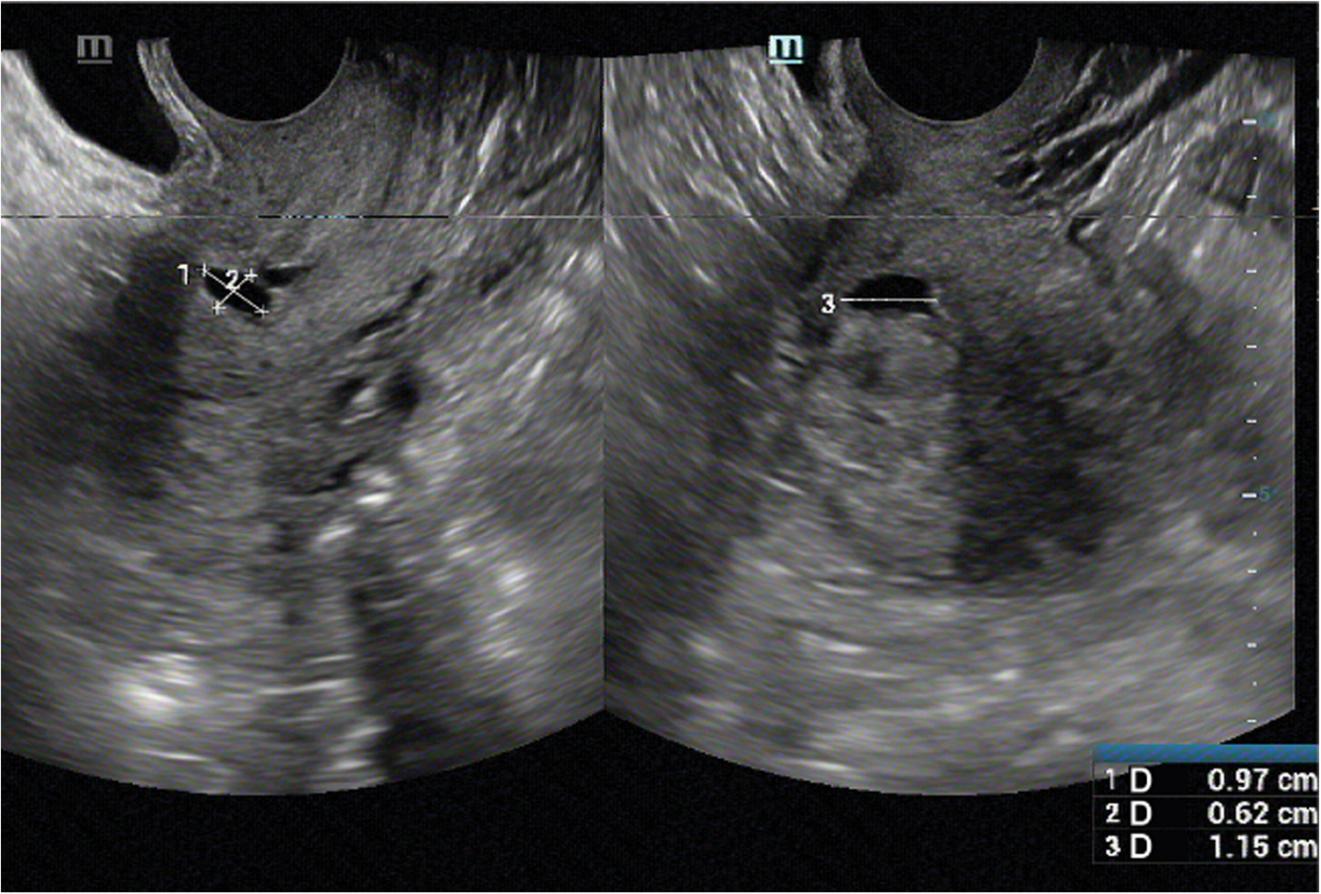
Ultrasound images of a cesarean scar defect. Size of scar defect (Length* Width* Depth):9.7* 11.5 *6.2 mm

The diagnosis of CSD was established by TVS and hysteroscopy. All TVS were performed by a team with three ultrasound operators, and sonograms display a triangular anechoic filling defect. We measured the length, width, depth and RMT of CSD (Fig. 1). The experienced gynecologists performed hysteroscopy which shows the uterine anterior wall defect at the isthmus, to identify the presence of CSD in the isthmic or cervical site. All ultrasound operators and gynecologists have undergone unified, standardized training.

All CSD patients in the current study were initially treated with the standard regimen at the time they first presented to the clinic (Drospirenone-Ethinyl Estradiol tablet -Yasmin, containing 3.0 mg of drospirenone and 0.03 mg of ethinylestradiol, continued for 3 cycles of 21 days). However, their symptoms had not improved and need to be treated in surgical treatment.

**Fig. 2:**
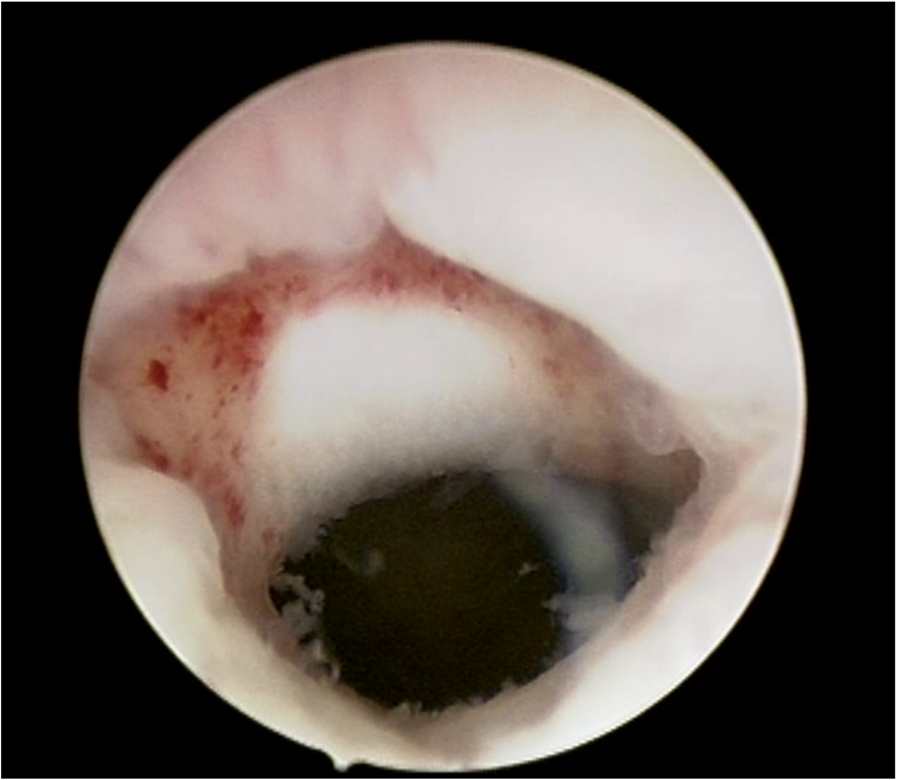
Hysteroscopic appearance of a cesarean scar defect

For each patient, decisive diagnoses repair of CSD was performed by a gynecologist follow the procedures as described below: under combined spinal and epidural anesthesia, patients were placed in a dorsal lithotomy position and the bladder was emptied. After sterile preparation and bladder catheterization, the cervix uteri was visualized using a vaginal retractor, and the anterior lip of the cervix was held with grasping forceps. A bipolar electrode (size: 9 mm) was then introduced under direct visualization via hysteroscopy (Olympus UES 40, Olympus, Japan). Physiological saline solution was used as a medium of uterine distension. After determining where the diverticulum was located, we performed a resection of inferior edges of the defect using a cutting loop. The bottom of the pouch was treated by use of aimed electrocauterization with a roller-ball 3-mm. At the same time, the endometrial tissue was taken for biopsy to rule out the presence of endometrial lesions, such as endometrial polyps, intimal hyperplasia, and endometrial cancer (Fig. 2).

### Data acquisition and patient follow-up

Baseline data from medical records contained patient demographics, surgical details, menstruation, and reproductive history. Length, width, depth, and RMT of the diverticulum will be registered through TVS. All patients were followed up in the 3rd and 6th month after surgery according to the established clinical pathways. Assessments about the improvement of CSD related symptoms (menstrual duration, menstrual volume, abdominal pain, and irregular bleeding) were mainly performed by a hospital visit. When follow-up assessments were not reported in the clinic medical records, telephone interviews were conducted by an obstetrician with at least 5 years of experience (Telephone interviews guide, see Additional file 1). The timing of surgery was defined as the interval between the first day of menstruation when blood starts to come out of the vagina and surgical treatment date.

### Outcome measures

The outcome was a postoperative improvement of CSD related symptoms. In this study, an improvement was defined as: (1) the postoperative menstrual duration shortened to < 7 days or 2 days shorter than the previous duration before surgery, or (2) alleviation of irregular bleeding, abdominal pain, and vaginal discomfort.

### Statistical analysis

Standard descriptive statistics were used, including percentages for discrete variables and mean ± SD for continuous variables. Categorical data were analyzed using the chi-square test, and continuous data using the independent samples t-test or Wilcoxon rank-sum test where appropriate. Logistic regression was used to evaluate prognostic factors of CSD recovery. The receiver-operating characteristics (ROC) curve was used to indicate the predictive role of the timing of surgery in CSD recovery. The area under curve (AUC) was calculated with its 95% CI. All reported *P* values are two-sided, and a *P* value of less than 0.05 was considered statistically significant. Statistical analysis was performed using SPSS 19.0.

## Results

### Patient characteristics

**Fig. 3:**
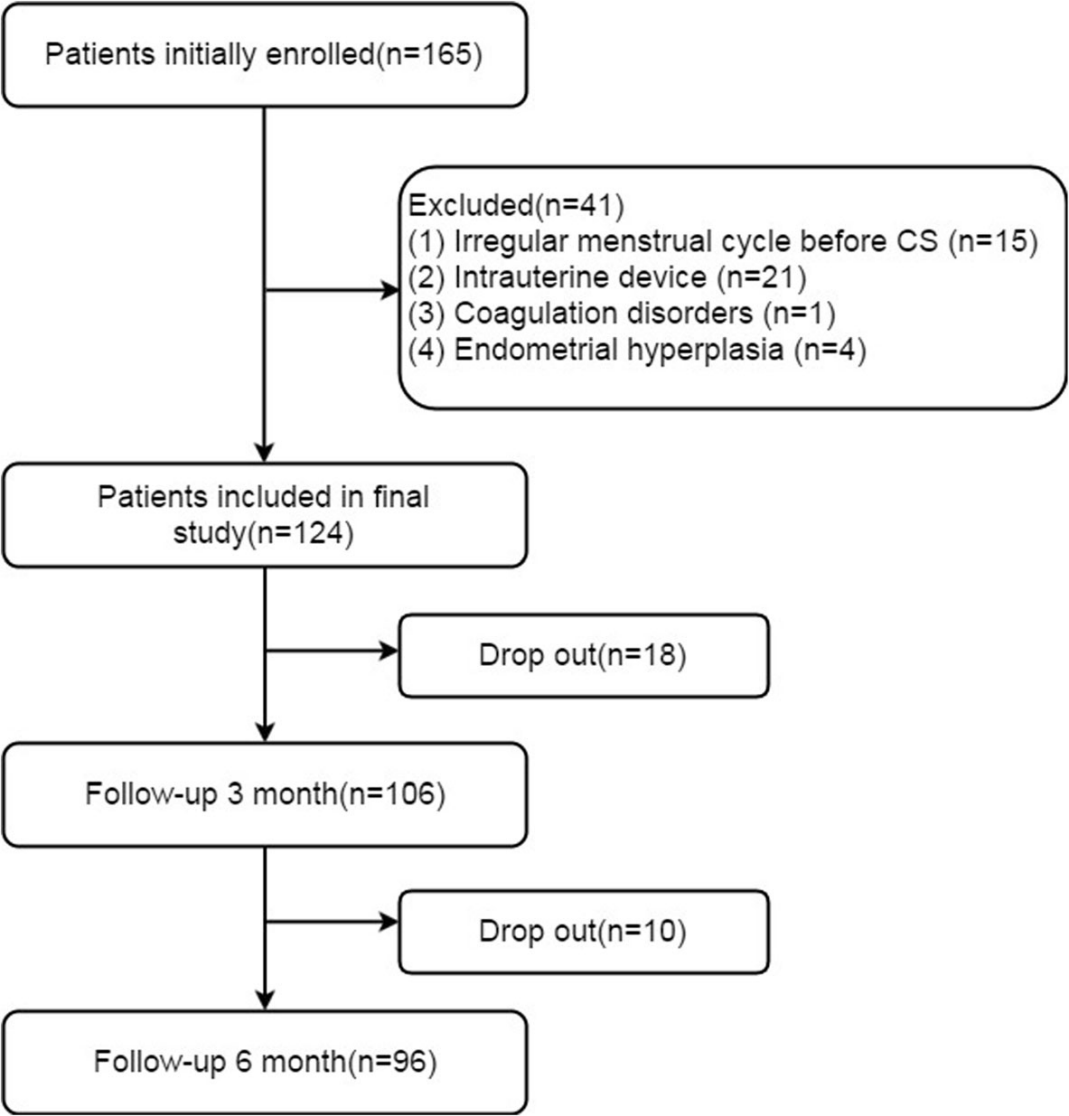
Flow diagram detailing the patients included in the retrospective analysis

As shown in the flowchart, 165 consecutively eligible patients with one surgeon were initially enrolled, and 124 patients were included in the final study. There were 18 and 10 patients were lost to follow-up at 3 and 6 months after CSD, respectively. (Fig. 3).

Baseline characteristics of the patients before surgery of CSD are shown in Table 1. The average age of the patients was 35.0 ± 5.0 years (range 25–48), the mean of menarche age was 13.6 ± 1.3 years, and the mean of duration between symptom onset and surgery was 3.0 ± 2.8 years. A total of 63(50.8%) patients had undergone ≥2 deliveries by CS. Doppler ultrasound data showed that the mean length, width, depth, and RMT of the scar defects before the operation were 9.7 ± 6.7 mm,8.1 ± 7.3 mm,8.7 ± 5.9 mm, and 4.9 ± 2.1 mm, respectively.

**Table 1:** Baseline demographics and clinical characteristics for patients between improvement and no improvement group

Characteristics	Total(*n* = 124)	3 months(*n* = 106)			6 months(*n* = 96)		
		Improvement	No Improvement	P_3m_^a^	Improvement	No Improvement	P_6m_^a^
Age (years)	35.0 ± 5.0	34.7 ± 4.8	35.4 ± 5.5	0.50	34.5 ± 4.7	35.4 ± 6.1	0.42
Age of menarche (years)	13.6 ± 1.3	13.5 ± 1.1	13.7 ± 1.3	0.51	13.5 ± 1.1	13.8 ± 1.4	0.29
Duration between symptom onset and surgery (years)	3.0 ± 2.8	3.2 ± 2.8	3.0 ± 3.1	0.77	2.9 ± 2.3	3.3 ± 3.6	0.52
Yeas between CS and surgery (years)	7.0 ± 3.7	7.3 ± 3.5	7.5 ± 4.2	0.80	6.8 ± 2.9	7.8 ± 4.3	0.18
Number of CS							
1	61 (49.2%)	23 (46.0%)	16 (28.6%)	0.04	31 (49.2%)	7 (21.2%)	0.004
≥ 2	63 (50.8%)	27 (54.0%)	40 (71.4%)		32 (50.8%)	26 (78.8%)	
Abortion times	1.0 ± 1.1	0.9 ± 1.0	1.2 ± 1.0	0.12	0.8 ± 0.9	1.4 ± 1.1	0.01
Surgical timing (days)	14.2 ± 5.5	12.0 ± 4.8	15.3 ± 5.8	0.002	11.7 ± 4.6	14.3 ± 5.8	0.03
Size of scar defects (mm)							
Length	9.7 ± 6.7	9.1 ± 8.3	9.5 ± 4.5	0.75	9.4 ± 7.8	9.3 ± 7.8	0.98
Width	8.1 ± 7.3	8.5 ± 9.3	7.6 ± 5.3	0.53	7.9 ± 7.8	6.7 ± 4.0	0.45
Depth	8.7 ± 5.9	8.8 ± 5.3	9.2 ± 6.1	0.78	8.9 ± 5.9	8.6 ± 5.7	0.78
RMT	4.9 ± 2.1	5.3 ± 2.3	4.9 ± 2.1	0.48	5.1 ± 2.3	4.9 ± 2.1	0.77
BMI (kg/m^2^)	21.3 ± 2.6	21.0 ± 2.4	21.5 ± 2.7	0.23	21.5 ± 3.1	21.0 ± 2.4	0.39
Hemoglobin(g/L)	119.0 ± 14.7	118.7 ± 16.0	119.3 ± 13.6	0.83	118.1 ± 17.9	119.4 ± 13.2	0.64
Anemia							
Yes	31 (25.0%)	11 (22.0%)	14 (25.0%)	0.94	13 (20.6%)	10 (30.3%)	0.22
No	93 (75.0%)	39 (78.0%)	42 (75.0%)		50 (79.4%)	23 (69.7%)	

*CS* caesarean section, *RMT* residual myometrial thickness

^a^mean ± SD for continuous variables and percentages for discrete variables

At the same time, we assessed the amount of blood loss and operation time of the patients during surgery, the mean of intraoperative blood loss and operative time were (12.94 ± 12.63) ml and (33.63 ± 6.87) min of 124 patients.

### The improvement after CSD surgical treatment

The proportion of patients who reported improvement of menstrual symptoms after CSD repair was 47.2% (50/106) within 3 months, and 65.6% (63/96) within 6 months. Compared with preoperative, the proportion of patients with more menstrual duration, much menstrual volume, and irregular bleeding decreased significantly at 3 months and 6 months after surgery(*P* < 0.05). A significant improvement in the irregular bleeding was observed in the 3, 6 months after surgery (*P* < 0.05). However, there was no significant difference in menstrual volume and abdominal pain between the two periods (*P* = 0.67, *P* = 0.07) (Table 2).

**Table 2:** Symptom improvement in patients with cesarean section diverticula 3 and 6 months after hysteroscopic resection (n = 96)

Characteristic	Before	3 months	6 months	P_B-3m_^a^	P_B-6m_^a^	P_3m–6m_^a^
Menstrual duration (day)	11.5 ± 4.0	8.8 ± 3.8	8.0 ± 3.7	<.001	<.001	0.14
≤7 (day)	16 (16.7%)	45 (46.9%)	60 (62.5%)	<.001	<.001	0.03
> 7 (day)	80 (83.3%)	51 (53.1%)	36 (37.5%)			
Menstrual volume (bleeding)						
Much	29 (30.2%)	16 (16.7%)	12 (12.5%)	0.03	0.002	0.67
Moderation	56 (58.3%)	73 (76.0%)	78 (81.3%)			
Litter	11 (11.5%)	7 (7.3%)	6 (6.3%)			
Abdominal pain						
Yes	23 (24.0%)	19 (19.8%)	10 (10.4%)	0.48	0.01	0.07
No	73 (76.0%)	77 (80.2%)	86 (89.6%)			
Irregular bleeding						
Yes	51 (53.1%)	27 (28.1%)	15 (15.6%)	<.001	<.001	0.04
No	45 (46.9%)	69 (71.9%)	81 (84.4%)			

^a^mean ± SD for continuous variables and percentages for discrete variables

### Comparison of clinical parameters between the improvement group and no improvement group at 3 months and 6 months after CSD

Table 1 also shows the characteristics of the improvement and no improvement groups in both 3 months and 6 months. These characteristics were generally similar in the two groups except the number of CS, abortion times, and timing of surgery. Patients who had undergone≥2 CS were more likely to have no improvement compared with those who had one CS at 3 months and 6 months (P_3m_ = 0.04, P_6m_ = 0.004), and lower times of abortions were associated with a higher chance of improvement at 6 months (P_6m_ = 0.01). The timing of surgery was significantly shorter in the improvement group when compared with that of the no improvement group within both 3 months and 6 months (P_3m_ = 0.002, P_6m_ = 0.03).

**Fig. 4:**
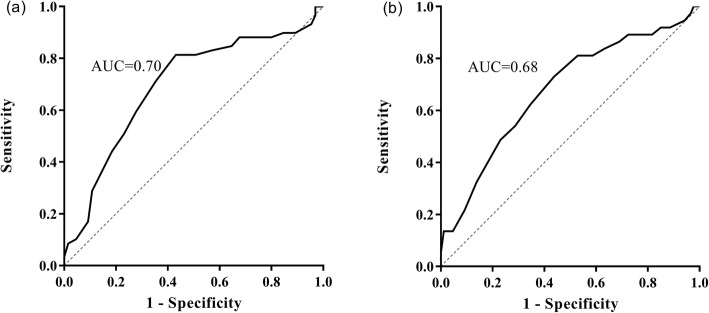
Receiver-operator characteristic (ROC) curve for the timing of surgery over post-operative improvement at 3 months (**a**) and 6 months (**b**)

On receiver-operating characteristic curve analyses, the timing of surgery had significant performance in predicting CSD at 3 months (AUC, 0.70; 95% CI, 0.60–0.79) and 6 months (AUC, 0.68; 95% CI, 0.57–0.78) post-operative improvement, as shown in Fig. 4.

### Prognostic factors of CSD recovery

Multivariable logistic regression showed that the postoperative improvement rate for the timing of surgery< 14 days group was significantly better than that for the group of ≥14 days group at 3 months (OR, 16.59; 95% CI, 2.62–104.90; *P* = 0.003), similar results were found for post-operative improvement at 6 months (OR, 15.51; 95% CI, 1.63–148.00; *P* = 0.02) (Table 3). Besides, the duration between symptom onset and surgery was shorter in the improvement group than in the no improvement group at 6 months (OR, 0.64; 95% CI, 0.41–0.99; *P* = 0.04). Patients with times of CS ≥ 2 had a lower rate of improvement after 6 months of CSD repair surgery compared with patients who underwent one CS (OR, 8.29; 95% CI, 1.05–65.75;P = 0.04).

**Table 3:** Prognostic factors for symptom improvement in patients with cesarean scar defect 3 and 6 months after hysteroscopic resection

Variables	3 months		6 months	
	Orrds Ratio (95%Cl)	P	Orrds Ratio (95%Cl)	P
Age (years)	1.12 (0.89–1.42)	0.36	1.15 (0.86–1.53)	0.36
Age of menarche (years)	0.67 (0.37–1.20)	0.17	0.61 (0.32–1.16)	0.13
Duration between symptom onset and surgery (years)	0.71 (0.46–1.08)	0.11	0.64 (0.41–0.99)	0.04
Age of CS (years)	1.26 (0.89–1.77)	0.19	1.10 (0.75–1.59)	0.66
Number of CS				
1	1.00		1.00	
≥ 2	3.27 (0.72–14.88)	0.13	8.29 (1.05–65.75)	0.04
Abortion number	0.76 (0.34–1.70)	0.50	0.53 (0.19–1.52)	0.24
Surgical timing (days)				
< 14	16.59 (2.62–104.90)	0.003	15.51 (1.63–148.00	0.02
≥ 14	1.00		1.00	
Length of defect (mm)	0.98 (0.84–1.13)	0.73	0.89 (0.74–1.06)	0.19
Width of defect (mm)	1.02 (0.91–1.14)	0.74	1.01 (0.90–1.29)	0.42
Depth of defect (mm)	0.94 (0.82–1.07)	0.36	1.04 (0.88–1.23)	0.65
RMT (mm)	0.94 (0.68–1.23)	0.57	0.76 (0.51–1.11)	0.16
BMI	0.82 (0.60–1.11)	0.20	1.39 (0.86–2.25)	0.18
Anemia				
Yes	1.00		1.00	
No	0.66 (0.12–3.72)	0.64	0.51 (0.06–4.40)	0.54

*CS* caesarean section, *RMT* residual myometrial thicknes

## Discussion

Owing to the increasing number of deliveries via CS worldwide, subsequent complications associated with CS, such as prolonged menstruation, irregular genital bleeding, and secondary infertility, have become a considerable concern and treatment for these complications has drawn more and more attention. The association between the diverticulum and bleeding disorders is gradually revealed [13].

Currently, the two main treatment options include hormonal therapy and surgical repair of the diverticulum [14]. Studies reported that CSD related menstrual bleeding disorders or cyclic pain do often not respond to hormonal therapies [8, 9]. Methods of operative repair of diverticulum include vaginal repair, laparoscopy, and hysteroscopy. The technique of defect repair through the hysteroscopy used in our clinic results in control of intermenstrual bleeding and pain control. One system review display that hysteroscopy is the most commonly reported approach for the revision of CSD and the existing evidence is inadequate to conclude that either hysteroscopy or laparoscopy is effective or superior to each other [7]. However, hysteroscopy may have a potential risk of decreased resistance of the residual myometrial tissue at the level of the repair and furthermore may lead to uterine rupture during subsequent pregnancy [15, 16].

We believed that the choice of the surgical approach is mainly based on the clinical features of patients [17]. In our population, all patients have no plans to conceive again and the residual myometrium thickness should not be less than 3 mm, given the anticipated risk on perforation or bladder injuries [11]. Indeed, we only resected the distal rim of the defect to prevent proximal resection could harm the strength of the cervix and may induce unneeded cervical incompetence. Concurrently superficial coagulation of vessels in the niche aims at reducing blood loss from these fragile vessels.

Under this premise, the cure rate of CSD repair was 47.2% after 3 months, and 65.6% after 6 months in our study. However, the cure rate of CSD repair reportedly ranges between 54 and 84%, [3] varying considerably depending on the study. For example, Fabres et al. showed that 84% of CSD patients (20/24) were successfully treated by hysteroscopic surgery after a follow-up of 24 months [18]. In the study of Wang et al., only 59.6% of patients (34/57) reported a postoperative improvement in symptoms after 3 months of surgery [19]. The difference is mainly due to the absence of universal assessment criteria for CSD symptom improvement. Furthermore, data from one study showed that the intraoperative blood loss, operative time of hysteroscopy resection were (10.1 ± 10.2 ml) and (20 ± 5.6 min), lower than vaginal repair(*P* < 0.05) and combined laparoscopic and hysteroscopy(P < 0.05) [20]. The results of our study are similar to the above (intraoperative blood loss, 12.89 ± 12.59 ml; operative time, 44.73 ± 17.12 min). We and others assume that hysteroscopic resection is more cost-effective. Moreover, in our study, no major complications, such as massive bleeding or uterine perforation, were encountered during surgery.

Our study confirms that a higher number of previous CS is associated with poorly improved symptoms. In the studies cited [21, 22], the numbers of scar defects and large scar defects increase as the number of CS increases, the ultrasound examiner found that the more CS, the more difficult it was to evaluate the individual scars, and the number of scars seen at ultrasound imaging did not always correspond to the number of Cesarean sections in women who had undergone more than one Cesarean section. Not all CSD is likely treated completely, and this might explain why patients who had undergone≥2 CS were more likely to have no improvement compared with those who had one CS.

Clear and broad vision to ensure the successful hysteroscopic surgery of CSD, effective removal of the lower margin of the uterine diverticulum and intimal tissue in the incision is essential, hypertrophic endometrium can affect the surgical field of vision [23], at the same time, the endometrium is thin in the first half of the menstrual cycle due to hormones. Therefore, surgery should be performed in the first half of the menstrual cycle to ensure a clear view of the operation. Obstetrics and Gynecology of Chinese Medical Association Branch proposed the norm about gynecological hysteroscopy, and it suggests that Hysteroscopic surgery should be selected in the early follicular phase of the implementation, 21 which is convenient for operation because of broad vision [24]. Our findings also confirm that poor outcomes were associated with a longer interval between the last menstrual period and the surgical treatment date (timing of surgery), this seems natural because the clear and broad vision of CSD surgery is likely to be an important factor affecting the improvement of postoperative symptoms. Taken together, these findings suggest that the timing of surgery may be an indicator of the incidence of incomplete relief of the performance of the menstrual cycle irregular menstruation after CSD repair.

The present study has several limitations. First, because our study was retrospective, CSD symptom relieve destination for patients was subject to selection bias and unmeasured confounding. This might include factors that determine the severity of CSD, technical factors and processes of surgery, and access to the Guangzhou Women and Children Medical Center, a tertiary obstetrics and gynecology hospital. Second, the small number size of the present study does not allow to extrapolate our findings to other population, and a proportion of patients were lost to follow-up at the 6th month, may lead to an underestimation of the improvement rate. Third, the parameter variables of CSD involved in our study were only the length, width, depth, and RMT, other parameters that may affect the prognosis are not fully considered. Final, the follow-up period (six months) might be too short to generalize our results to longer-term outcomes after CSD. But one recent study suggested that postoperative menstruation and imaging data did not differ markedly between 3 and 6 months after surgery, suggesting that follow-up at 6 months represents an adequate endpoint for evaluating the effectiveness of surgery [25].

## Conclusion

Hysteroscopic repair can improve the symptoms of CSD patients and it has the least invasive and quick recovery. However, hysteroscopic repair may be more suitable for those who do not have the willingness to give birth and want to improve their symptoms. It is also more suitable for people with thicker RMT (≥3 mm). The suitable timing of surgery (timing of surgery< 14 days) and the history of CS may be important factors affecting postoperative prognosis.

## Supplementary information

**Additional file 1.** Questionnaire on the treatment of C-section diverticula with hysteroscopic resection in women without childbearing intention.

## Data Availability

The datasets generated and/or analyzed during the current study are not publicly available due to potential for individual and organizational privacy to be compromised. Reasonable requests for parts of the data will be considered by the corresponding author.

## Abbreviations

AUC: Area under curve
CS: Caesarean section
CSD: Cesarean section diverticulum
TVS: Transvaginal ultrasonography
RMT: Residual myometrial thickness
ROC: Receiver-operating characteristics
