# Supplementary material for: Treatment of C-section diverticula with hysteroscopic resection in women without childbearing intention: a retrospective cohort study
Source: BMC Womens Health. 2020 Apr 21;20:75. doi: 10.1186/s12905-020-00928-4 (PMC7171842; doi:10.1186/s12905-020-00928-4)
Supplement: Supplementary file 1 — Additional file 1. Questionnaire on the treatment of C-section diverticula with hysteroscopic resection in women without childbearing intention. [file 12905_2020_928_MOESM1_ESM.pdf]

Questionnaire on the treatment of C-section diverticula with hysteroscopic resection in women without childbearing intention

Oral informed consent

Project Name： Treatment of C-section diverticula with hysteroscopic resection in women without childbearing intention

Project Number: No. GO-2017-017

Informed Consent Version Number: 02, 20171206

Research institute: Guangzhou Women and Children's Medical Center

Main researcher: Dr Huiying Liang, Dr Hui Shi

You will be invited to participate in a clinical study. This talk can provide some information to help you decide whether to participate in this clinical study. Your participation in this study is voluntary and this study has been reviewed by the ethics committee of Guangzhou Women and Children's Medical Center.

In this study, we aim to describe the improvement after hysteroscopic resection of cesarean section diverticula (CSD) in women without childbearing intention, and to explore the variables associated with poor prognosis.

If you agree, we will extract your current medical data from the electronic medical record, including ultrasound data. No biological specimens need to be collected during the process.

Benefit: analysis of your diagnosis and treatment data can provide necessary advice for your treatment.

All information is confidential and identifiable information will not be disclosed to members outside the research team, unless your permission is obtained. Your personal information will not be disclosed when the results of this research are published.

You can choose not to participate in this research, your data will not be included in the research results, and any of your medical treatment and rights will not be affected by this. You can always know the information and research progress related to this project. If you have any questions, please call and let me know. My phone number is 020-38857692.

Does the patient agree to participate? ☐ **Yes** ☐ **No**

Signature of the doctor:

Data:

Hospital number________ **ID_________**

Part I. General Patients (Available from electronic medical records)

A1. Name: _______

A2. Age (year): _______

A3. Nation: __________ (1) Han nationality (2) Others

A4. Education： _____ (1) Primary school (2) Middle school (3) Senior school (4) College or above

A5. Marital status： ____ (1) Unmarried (2) Married (3) Divorced (4) Spouse

A6. Are you willing to continue having children?_______(1) Yes (2) No

Part II. Patient's Menstrual History and Motherhood History (Available from electronic medical records)

B1. Age of menarche (year):______

Average menstrual duration before cesarean section (day):______

B2. Up until now, how many times has the patient been pregnant?____

How many times has the patient delivered? ____

How many times did the patient miscarry?____

How many of them are aborted?____

B3. How many times did the patient have a cesarean section?______ Among them：

Date of the first cesarean section:__________

Uterine incision： ____(1) Upper anterior uterine (2) Lower anterior uterine (3) Corpus uteri

Sex of the first child:_____ (1)Boy (2) girl；

Birth weight(g)： ______ Birth length (cm)________

Whether the following complications were diagnosed during the first pregnancy?______ （Multiple options）

(1) Gestational hypertension (2) Gestational diabetes mellitus (3) premature rupture of fetal membranes

(4) Placenta praevia (5) Placental abruption

Date of the second cesarean section:__________

Uterine incision： ____(1) Upper anterior uterine (2) Lower anterior uterine (3) Corpus uteri

Sex of the first child:___ (1)Boy (2) girl；

Birth weight(g)： ______ Birth length (cm)________

Whether the following complications were diagnosed during the second pregnancy?______ （Multiple options）

(1) Gestational hypertension (2) Gestational diabetes mellitus (3) premature rupture of fetal membranes

(4) Placenta praevia (5) Placental abruption

B4. Whether the patient has a history of premature birth?____ (1)Yes (2)No ；

Whether the patient has a stillbirth history?____ (1) Yes (2) No

Part III. Disease and treatment of patients with uterine diverticulum (Available from electronic medical records)

C1. When did clinical symptoms appear?___________

What are the clinical symptoms? ______ （Multiple options）

(1) Menstrual period extension (2) Increased menstrual flow (3) Back and lower abdomen pain (4) Irregular bleeding

(5) Fatigue (6) Others__________

Average menstrual duration before surgical (day)______

Menstrual volume before surgical：______ (1) Much (2) Moderation (3) Litter

Do you have abdominal pain?______ (1) Yes (2) No

Do you have irregular bleeding?______ (1) Yes (2) No

C2. When is the diagnosis of CSD? _______

Location of CSD： _______

Length of CSD (cm)_______. Width of CSD (cm)_______. Depth of CSD (cm)_______.

Residual myometrial thickness (RMT) of CSD (cm)_______.

C3. Date of surgery:____

Intraoperative blood loss(ml)： _______

operative duration(min)： _______

Part IV. Patient Prognosis (Available from electronic medical records or telephone follow-up)

D1. 3 months after operation:

Average menstrual duration (day)______

Menstrual volume before surgical：______ (1) Much (2) Moderation (3) Litter

Do you have abdominal pain?______ (1) Yes (2) No

Do you have irregular bleeding?______ (1) Yes (2) No

D2. 6 months after operation:

Average menstrual duration (day)______

Menstrual volume before surgical：______ (1) Much (2) Moderation (3) Litter

Do you have abdominal pain?______ (1) Yes (2) No

Do you have irregular bleeding?______ (1) Yes (2) No

Thank you for your participation!
